# A structure-based mechanism for displacement of the HEXIM adapter from 7SK small nuclear RNA

**DOI:** 10.1038/s42003-022-03734-w

**Published:** 2022-08-15

**Authors:** Vincent V. Pham, Michael Gao, Jennifer L. Meagher, Janet L. Smith, Victoria M. D’Souza

**Affiliations:** 1 Department of Molecular and Cellular Biology, Harvard University, Cambridge, MA 02138 USA; 2 Life Sciences Institute, University of Michigan, Ann Arbor, MI 48109 USA; 3 Department of Biological Chemistry, University of Michigan, Ann Arbor, MI 48109 USA

**Keywords:** Solution-state NMR, Transcriptional regulatory elements

## Abstract

Productive transcriptional elongation of many cellular and viral mRNAs requires transcriptional factors to extract pTEFb from the 7SK snRNP by modulating the association between HEXIM and 7SK snRNA. In HIV-1, Tat binds to 7SK by displacing HEXIM. However, without the structure of the 7SK-HEXIM complex, the constraints that must be overcome for displacement remain unknown. Furthermore, while structure details of the Tat^NL4-3^-7SK complex have been elucidated, it is unclear how subtypes with more HEXIM-like Tat sequences accomplish displacement. Here we report the structures of HEXIM, Tat^G^, and Tat^Fin^ arginine rich motifs in complex with the apical stemloop-1 of 7SK. While most interactions between 7SK with HEXIM and Tat are similar, critical differences exist that guide function. First, the conformational plasticity of 7SK enables the formation of three different base pair configurations at a critical remodeling site, which allows for the modulation required for HEXIM binding and its subsequent displacement by Tat. Furthermore, the specific sequence variations observed in various Tat subtypes all converge on remodeling 7SK at this region. Second, we show that HEXIM primes its own displacement by causing specific local destabilization upon binding — a feature that is then exploited by Tat to bind 7SK more efficiently.

## Abstract (web summary)

Here the structure of the HEXIM arginine rich motif in complex with the stemloop-1 of 7SK snRNA is determined, and insights are provided into how the HIV protein Tat overcomes the structural constraints required to displace HEXIM, an event required for the extraction of pTEFb during transcriptional elongation.

## Introduction

Transcription of all class II genes is a highly regulated process within cells. Shortly after promoter clearance, RNA Polymerase II is inhibited by negative elongation factors^1–5^. Release from this stalled state requires all components to be phosphorylated by the positive elongation factor pTEFb, a heterodimeric complex consisting of Cyclin T1 and the cyclin-dependent kinase Cdk9^6–12^. However, most of the pTEFb is kept catalytically inactive in the nucleus by the 7SK small nuclear ribonucleoprotein (7SK snRNP) through its interactions with the HEXIM adapter protein and the 5’ stemloop-1 of the 7SK RNA^13–23^ (7SK-SL1). Thus, productive transcriptional elongation of many genes requires transcriptional factors to extract pTEFb from the 7SK snRNP—a process that involves manipulating the interaction between HEXIM and 7SK. This association between 7SK and HEXIM tightly controls the balance between active and inactive pTEFb, and dysregulation of this interaction can have serious biological consequences, including cardiac hypertrophy and breast and pancreatic cancers^24–27^. Furthermore, as many viruses rely on the host transcriptional machinery to produce mRNA and genomes, they have also evolved mechanisms to capture pTEFb^28–30^. One such unique case is the human immunodeficiency virus (HIV), which utilizes the viral Tat protein to extract pTEFb by binding to the same region of 7SK as HEXIM and directly displacing it^30–34^. Structural insights into the consequence of HEXIM binding to 7SK and how positive transcriptional factors like Tat compete with it are therefore important for understanding HEXIM’s potency as a critical negative regulator.

To date, two HEXIM proteins have been identified that can carry out the same function and both bind 7SK with identical regions of their Arginine-Rich Motifs (ARMs) (residues 151–159 in HEXIM1 and 89–97 in HEXIM2)^30,35–38^. Although HEXIM binds 7SK as a dimer, only one ARM directly contacts 7SK by engaging the apical region of stemloop-1 (G_26_ to C_85_, 7SK-SL1^apical^)^38–44^. Both in vitro and in vivo studies have shown that this represents the sole interaction between the two molecules that must be modulated to release pTEFb^37–39,41,42^.

Our previous work showed that 7SK-SL1^apical^ is enriched in arginine sandwich motifs (ASMs)^45^. ASMs are defined by two nucleotides that stack in a manner that allows for intercalation of arginine guanidinium moieties between the aromatic rings of the bases^45–51^. While a bulge pyrimidine forms the cap by engaging in a triple-base interaction with an n + 2 base pair in the stem, a Watson–Crick base-paired nucleotide preceding the bulge forms the base of the interaction. In the free 7SK-SL1^apical^, three such bulges fold into preformed arginine sandwich motifs (ASM_1_, ASM_2_, and ASM_4_) poised for arginine guanidinium moieties to dock into them. A fourth bulge folds into a pseudo configuration (pseudo-ASM_3_) where U_40_ can form a triple-base interaction with the A_43_-U_66_ base pair to form the cap, but the base of the sandwich is sequestered in a reverse Hoogsteen interaction, excluding it from use as a classical ASM. Our work also showed that HIV-1 Tat NL4-3 (Tat^NL4-3^) uses its arginine-rich motif to intercalate arginines not only into the three preformed ASMs, but also to remodel the pseudo-ASM into a classical ASM^45^. This structural remodeling of pseudo-ASM_3_ is a key mechanism through which Tat displaces HEXIM.

However, without the structure of the HEXIM:7SK-SL1^apical^ interaction, it is currently unclear what structural constraints Tat would need to overcome to access pTEFb. Furthermore, while the Tat ARM is highly conserved, sequence variations exist in different strains that allow for HEXIM displacement. For example, the ARM of Tat Finland (Tat^Fin^; KR_52_KHRRR) differs from HEXIM (KK_151_KHRRR) by only a single amino acid and would lack one of the ASM interactions from the previously described Tat NL4-3 strain (KR_52_RQRRR). Additionally, while Tat subtype G (Tat^G^; KR_52_R_53_HRRR) has an equivalent number of arginines as Tat^NL4-3^, the critical linker sequence connecting the ASM_3_/ASM_4_ and ASM_1_/ASM_2_ interactions is the same as in HEXIM. In this study, we present the structure of the 7SK-SL1^apical^ in complex with the HEXIM, Tat^Fin^, and Tat^G^ ARMs. Despite sequence variations, the structures show deep major groove intercalations of all ARMs, albeit with differential interactions with pseudo-ASM_3_ and ASM_4_. Furthermore, we show that HEXIM causes local destabilization of ASM_4_, enhancing Tat’s affinity for 7SK. These studies thus uncover a feature in which HEXIM facilitates its own displacement by increasing conformational sampling, which may be a more general mechanism of pTEFb capture.

## Results

### Comparative binding affinities of HEXIM and Tat to 7SK

**Fig. 1:**
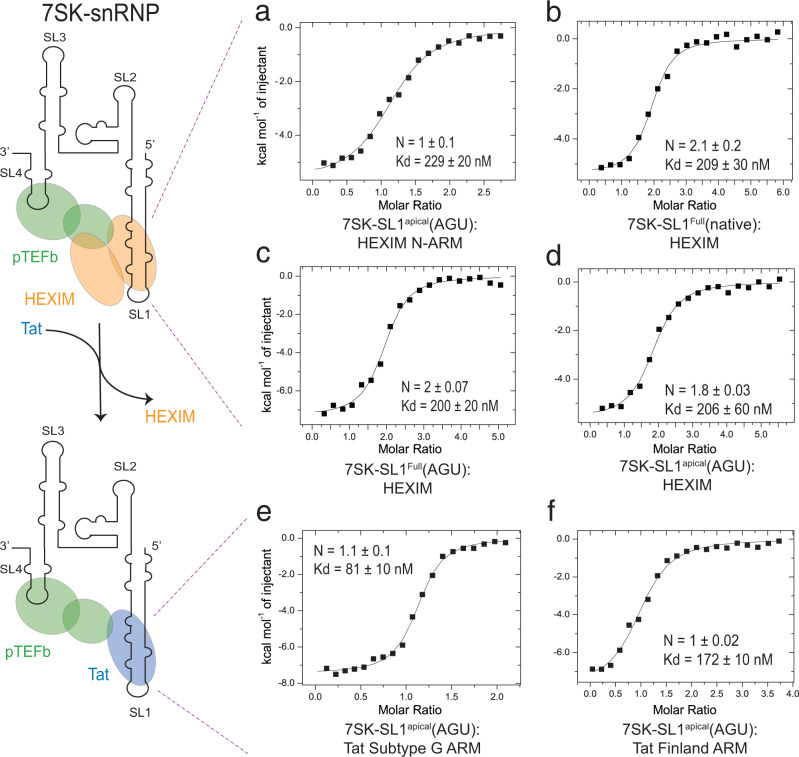
Characterization of HEXIM and Tat binding to 7SK. (Left) Cartoon representation of the HEXIM dimer and pTEFb heterodimer binding to the 7SK snRNP (top left). Upon introduction of Tat, HEXIM is displaced from the snRNP (bottom left). Not depicted are MEPCE and LARP7. Representative ITC data for HEXIM^N-ARM^ binding to 7SK-SL1^apical-AGU^ (G_26_-C_85_) (**a**) compared to full-length HEXIM1 bound to 7SK-SL1^Full^ (G_1_-C_108_) with a wild-type loop (**b**) or an AGU triloop (**c**) and full-length HEXIM1 binding to 7SK-SL1^apical-AGU^ (**d**) all show similar binding affinities, indicating that the loop does not play a significant role in dimeric HEXIM binding and that the HEXIM^N-ARM^:7SK-SL1^apical-AGU^ complex represents the minimal binding interaction. Representative ITC traces of the Tat^G^ (**e**) and Tat^Fin^ (**f**) ARMs into 7SK-SL1^apical-AGU^ show an ~2.8 and 1.3-fold increased binding affinity compared to HEXIM^N-ARM^, respectively. All reported values are for *n* = 3 replicates.

As a first step toward identifying the comparative thermodynamic properties of 7SK recognition between HEXIM and Tat, we performed binding studies using isothermal titration calorimetry (ITC). ITC traces of the ARMs into 7SK-SL1^apical-AGU^ produce significant nonspecific heats of binding as previously observed^52^. In a previous study by Brillet et al., high salt conditions (0.5 M NaCl) were used to abrogate such nonspecific interactions that stem from charge-charge interactions between the positively charged peptides and the negative RNA backbone^52^. While such a strategy is commonly used, it is not ideal for this system as the structuring of ASMs in 7SK is highly sensitive to ionic conditions and folds only around physiological salt conditions (Supplementary Figs. 1, 2)^45^. Therefore, to subtract the nonspecific heats of binding, we designed a control construct that lacks all ASMs (7SK-SL1^apicalΔASM^). Indeed, the heats obtained from peptide titrations into this control construct completely accounted for the nonspecific heats, the subtraction of which allowed for experimental baselines to approach zero at saturation (Supplementary Fig. 2b). Titration of the N-terminal ARM residues of HEXIM (R_146_QLGKKKHRRR_156_; HEXIM^N-ARM^) into 7SK-SL1^apical-AGU^ containing an AGU triloop engineered to prevent low levels of dimerization gave a *K*_d_ of 229 ± 20 nM (*N* = 1 ± 0.1; Fig. 1a). The redesign of the previously used GAGA tetraloop^45^ to an AGU triloop was done to prevent weak association between the tyrosine in the peptide and the tetraloop. Nevertheless, while the affinities obtained by AGU-triloop are 2 to 3-fold weaker, the relative difference between HEXIM and Tat are similar (see below).

To confirm that interactions do not extend to the loop and are represented by these minimal constructs, we performed studies with full-length HEXIM into full-length 7SK-SL1^Full^ (G_1_-C_108_), 7SK-SL1^Full-AGU^, and 7SK-SL1^apical-AGU^, all of which give rise to similar *K*_d_s (209 ± 30 nM, *K*_d_ = 200 ± 20 nM, and *K*_d_ = 206 ± 60 nM, respectively) and bound expectedly as dimers (*N* = 2.1 ± 0.2, *N* = 2 ± 0.07, and *N* = 1.8 ± 0.03, respectively; Fig. 1b–d). Furthermore, NMR studies comparing full-length dimeric HEXIM1:7SK-SL1^apical-AGU^ and HEXIM^N-ARM^:7SK-SL1^apical-AGU^ complexes show that binding of either full-length HEXIM or the N-ARM gives rise to the same chemical shifts in 7SK-SL1^apical-AGU^, indicating that the HEXIM^N-ARM^:7SK-SL1^apical-AGU^ interaction represents the biologically relevant mode of HEXIM binding to 7SK (Supplementary Fig. 2).

Our previous work showed that the Tat^NL4-3^ (KR_52_RQRRR) ARM represents the interaction domain between Tat and 7SK-SL1^apical^ and has an approximately two-fold increased affinity over the HEXIM^N-ARM^, which provides an explanation for HEXIM displacement^45^. ITC traces show that Tat Subtype G’s ARM (KR_52_RHRRR), which also has two N-terminal arginines, binds 7SK-SL1^apical-AGU^ with a *K*_d_ of 81 ± 10 nM (*N* = 1.1 ± 0.1; Fig. 1e), which is an approximately 2.8-fold increased binding affinity over HEXIM^N-ARM^ (Supplementary Table 1). On the other hand, Tat Finland’s ARM (KR_52_KHRRR), despite having an additional N-terminal arginine compared to the HEXIM^N-ARM^ (R52 and K151, respectively), does not have a statistically significant increase in binding affinity over HEXIM^N-ARM^ (*K*_d_ of 172 ± 10 nM, *N* = 1 ± 0.02; Fig. 1f). Overall, these results highlight the need for understanding the HEXIM-bound 7SK; while the increased Tat^G^ affinity would allow for HEXIM displacement, it is unclear how Tat^Fin^ can achieve the same biological output.

### Preformed configurations of ASM_1_ and ASM_2_ provide a common mode of interaction with C-terminal arginines

**Fig. 2:**
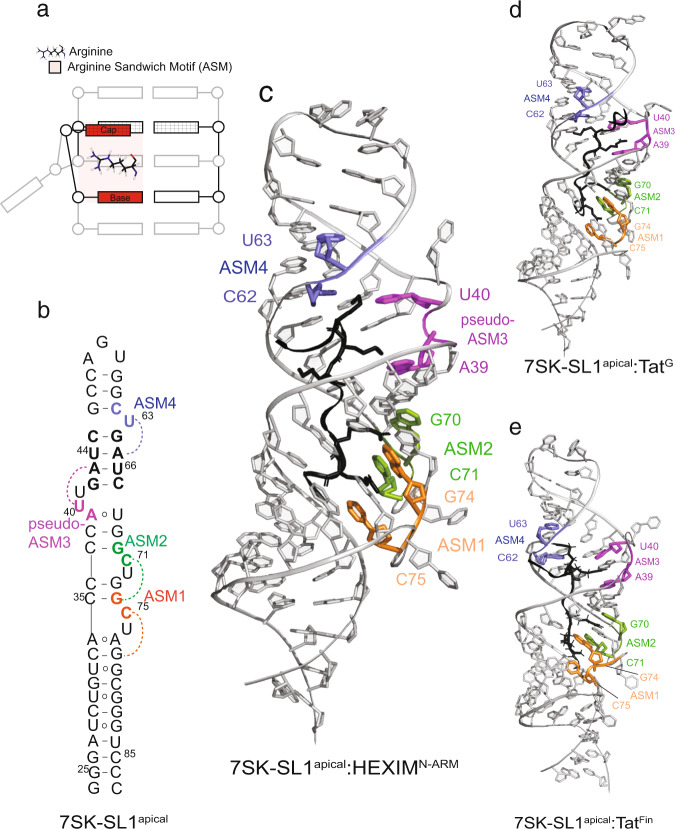
7SK-SL1^apical^ in complex with HEXIM^N-ARM^, Tat^Fin^, and Tat^G^ ARMs. **a** Cartoon depicting an arginine sandwich motif. **b** Secondary structure of free 7SK-SL1^apical-AGU^ with a modified AGU triloop. The base and cap residues forming ASM_1_, ASM_2_, pseudo-ASM_3_, and ASM_4_ are colored in orange, green, magenta, and blue, respectively. Dashed arcs represent triple-base interactions from the bulge to the stem, giving rise to the caps of the sandwiches. Representative NMR structures of 7SK-SL1^apical-AGU^ bound to (**c**) HEXIM^N-ARM^, (**d**) Tat^Fin^, and (**e**) Tat^G^ show engagement with all ASMs.

To understand how the HEXIM^N-ARM^ and the various Tat ARMs interact with 7SK-SL1^apical-AGU^, we utilized a combination of small-angle X-ray scattering (SAXS) and NMR. All reconstructed ab initio SAXS envelopes showed no major overall global changes between peptide-bound and free 7SK-SL1^apical-AGU^ (Supplementary Fig. 3). Numerous intermolecular NOEs place both HEXIM and Tat arginine-rich motifs into the major groove of the RNA and allow us to define their interactions with all ASM regions. Base pairs in the lower part of the stemloop below the G_79_-U_32_ base pair, as well as the CAGUG pentaloop do not give any intermolecular NOEs, indicating that the interactions are contained within a single turn of the helix (Fig. 2 and Table 1).

**Table 1:** NMR and refinement statistics for HEXIM, Tat^Fin^, and Tat^G^ ARMs in complex with 7SK-SL1^apical-AGU^.

	HEXIM	Tat^Fin^	Tat^G^
**NMR distance and dihedral constraints**			
Distance constraints			
Total NOE	584	589	588
Intra-residue	240	240	240
Inter-residue	344	349	348
Sequential (|*i* – *j* | = 1)	156	156	156
Medium-range (|*i* – *j* | < 4)	21	20	20
Long-range (|*i* – *j* | > 5)	167	173	172
Intermolecular	42	49	48
Hydrogen bonds	154	157	163
Total dihedral angle restraints	376	376	376
**Structure statistics**			
Violations (mean and s.d.)	323 ± 14	342 ± 14	348 ± 17
Distance constraints (Å)	0.28 ± 0.007	0.28 ± 0.01	0.26 ± 0.007
Dihedral angle constraints (°)	0.22 ± 0.05	0.16 ± 0.05	0.31 ± 0.08
Max. dihedral angle violation (°)	6.12 ± 1.01	7.89 ± 2.10	14.6 ± 1.96
Max. distance constraint violation (Å)	1.69 ± 0.13	1.62 ± 0.16	2.15 ± 0.28
Deviations from idealized geometry			
Bond lengths (Å)	0.006	0.006	0.006
Bond angles (°)	1.04 ± 0.009	1.06 ± 0.02	1.06 ± 0.005
Impropers (°)	0.68 ± 0.02	0.79 ± 0.06	0.94 ± 0.03
Average pairwise r.m.s. deviation* (Å)			
Heavy**	1.55 ± 0.32	0.59 ± 0.06	2.25 ± 0.43
Backbone**	0.44 ± 0.08	0.54 ± 0.06	0.38 ± 0.05

*Pairwise r.m.s. deviation was calculated among ten refined structures.

** These are residues 24:87 for the RNA and 150:157 for the peptides.

In the free 7SK-SL1^apical-AGU^, ASM_1_ and ASM_2_ are placed in tandem orientation, and upon titration of the various ARMs, all expected NOEs for such configurations are retained. Unlike a typical ASM where the following nucleotide after the bulge is in a canonical Watson–Crick base pair, in ASM_1_, the residue A_77_ is configured into an A_34_-A_77_ base pair. A NOE from the A_77_ H8 proton to the H1′ of C_75_ positions this residue under the C_75_ cap (Supplementary Fig. 4). This confirms a planar orientation of C_75_ with the C_33_–G_78_ base pair and configures A_77_ in such a way that it is perfectly positioned to interact with the guanidinium moiety of R156 in HEXIM^N-ARM^ and R57 in Tat^Fin^ and Tat^G^, which intercalate between C_75_ and G_74_ in a manner identical to canonical ASMs (Supplementary Figs. 4–7).

**Fig. 3:**
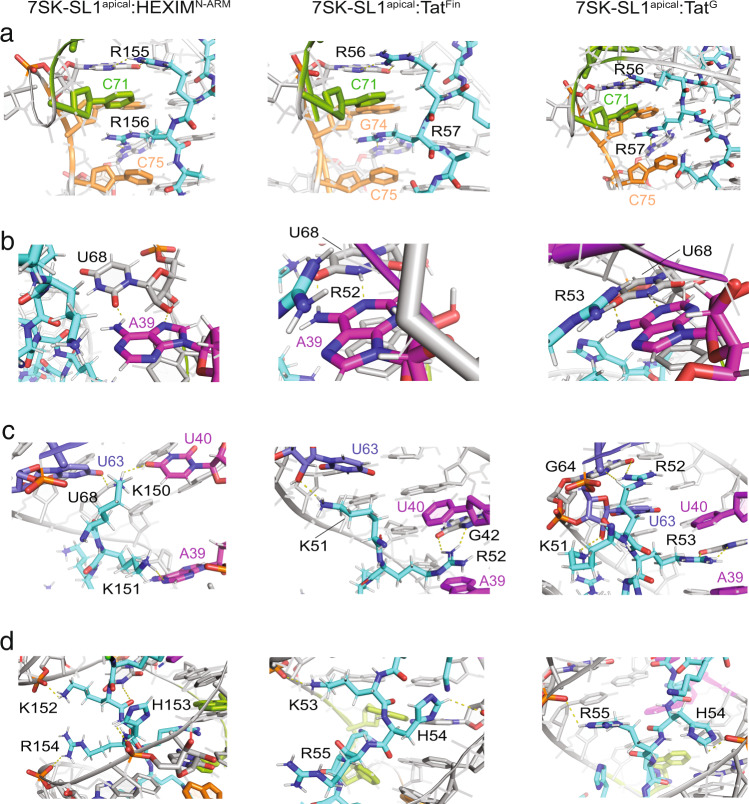
Details of intermolecular interactions between HEXIM^N-ARM^, Tat^Fin^, and Tat^G^ ARMs with 7SK-SL1^apical^ and the rearrangement of the U_68_-A_39_ base pair. **a** C-terminal arginines of all ARMs dock into ASM_1_ (orange) and ASM_2_ (green) with identical tertiary structures. **b** The U_68_-A_39_ base pair rearranges into a cis-Hoogsteen/sugar interaction upon HEXIM binding (left) while Tat^Fin^ (middle) and Tat^G^ (right) both remodel ASM_3_ (magenta) by rearranging the U_68_-A_39_ base pair into a Watson–Crick interaction. **c** K150 and K151 in HEXIM (left), R52 and K51 in Tat^Fin^ (middle), and K51, R52, and R53 in Tat^G^ (right) interact with the apical ASMs. **d** Spacer residues between the ASM_1_/ASM_2_ and ASM_3_/ASM_4_ regions are positioned near ASM_1_ and ASM_2_. In the case of Tat^Fin^ (middle), K53 also acts as a spacer residue to allow for the remodeling of ASM_3_ by R52.

Similarly, in ASM_2_, the C_71_^+^ base also retains its protonation at the N3 position, as evidenced by a downfield shift of the N4 amino protons (Supplementary Fig. 8). The guanidinium moiety of R155 in HEXIM^N-ARM^ and R56 of Tat^Fin^ and Tat^G^ interact with G_73_ by intercalating between the C_71_^+^ cap and G_70_ base of the motif (Supplementary Figs. 5–7). Additionally, intermolecular NOEs from the aromatic protons of the C_75_ and C_71_^+^ caps and the G_74_ and G_70_ bases of ASM_1_ and ASM_2_ to the Hγ and the Hδ protons confirm that consecutive arginines R156 and R155 interact in a ladder-like configuration with the tandem performed motifs ASM_1_ and ASM_2_, respectively (Fig. 3a and Supplementary Fig. 6c). Such NOEs are also observed in both the Tat^Fin^ and the Tat^G^-bound complexes, confirming the similar placement of the C-terminal R57 and R56 into the tandem ASM_1_ and ASM_2_, respectively (Fig. 3a and Supplementary Fig. 7a, b, d, e). Taken together, the structures reveal a common mode of interaction between the non-varying C-terminal arginines and the tandem ASMs.

### Rearrangement of pseudo-ASM_3_ allows for HEXIM N-terminal interactions

In the free 7SK-SL1^apical-AGU^, pseudo-ASM_3_ and ASM_4_ adopt a pseudo-symmetrical architecture where the two motifs are spatially opposed. Upon HEXIM^N-ARM^ binding, the pseudo-ASM_3_ maintains its U_40_:A_43_-U_66_ triple-base interaction although the base of the sandwich, A_39_, rearranges from a reverse Hoogsteen interaction with U_68_ into a cis-Hoogsteen/sugar interaction, giving rise to an alternate pseudo configuration. (Fig. 3b). This is evidenced both by NOEs from the U_68_ imino proton to the A_39_ amino protons and NOEs from the U_68_ H2′ and H3′ protons to the A_39_ H8 proton (Supplementary Fig. 8b). This frees up the U_68_ imino proton to engage the backbone carbonyl of K152 while simultaneously bringing the N1 proton acceptor of A_39_ into the major groove to hydrogen-bond with the side chain Hε protons of K151 (Fig. 3c). Thus, both K151 and 152 can enter deep into the major groove by remodeling the pseudo-ASM_3_.

The amino side chain of K151 is within hydrogen-bonding distance of the A_39_ N1 nitrogen as evidenced by NOEs from the K151 Hγ and Hβ protons to the C_37_ H6 and H5 protons, respectively, and from the K151 Hε protons to the C_38_ H6 and H5 protons (Fig. 3c and Supplementary Fig. 6f). Additionally, NOEs between the K152 Hβ protons with the U_68_ H5 proton, the K152 Hδ protons with the C_67_ and U_66_ H5 protons, and the K152 Hε protons with the C_67_ H5 and H6 protons position the amino side chain of K152 within hydrogen-bonding distance of the C_67_ backbone (Fig. 3c and Supplementary Fig. 6e, f). This gives rise to a forked configuration of the two lysines, orienting the side chain amino groups towards the phosphate backbones on opposite ends of the groove.

Unlike the other three ASMs, where the NOEs clearly define a single predominant structural configuration as described above, multiple dynamic states exist for ASM_4_ (see below). In the most abundant form, the preformed nature found in the free state is retained as evidenced by a direct imino-to-imino connectivity between U_44_ and U_63_ along with maintenance of the G_46_–C_62_ Watson–Crick base pair (Supplementary Fig. 8c). In fact, this interaction is stabilized by K150, which displays NOEs between the Hε protons with the U_63_ and the U_40_ H5 protons, positioning the amino side chain within hydrogen-bonding distance of the O4 atoms of both U_63_ and U_40_ (Supplementary Fig. 6d). Additional intermolecular interactions between the U_40_ H5 proton and the U_63_ H5 and H1′ protons with the K150 Hδ, Hγ, and Hβ protons places K150 directly under the U_63_ cap of ASM_4_ (Supplementary Fig. 6d, e). Taken together, these data show that despite the lack of arginines, the lysine-rich N-terminus of HEXIM^N-ARM^ can be accommodated by 7SK: the Watson–Crick face of A_39_ turns from the minor into the major groove to interact with K151 and 152, which then positions K150 to interact with the oxygen-rich environment of the U_63_ and U_40_ caps.

### Conformational plasticity of the ASM_3_/ASM_4_ region provides differential mode of interactions with N-terminal and spacer residues

Our previous study showed that Tat^NL4-3^ displaces HEXIM by remodeling the pseudo-ASM_3_ into a canonical ASM_3_ to allow for arginine intercalation^45^. Furthermore, an additional arginine docks into the preformed ASM_4_. While the mechanism of remodeling pseudo-ASM_3_ is conserved upon binding of both Tat^Fin^ and Tat^G^ ARMs (Fig. 3b, f and Supplementary Fig. 6), both the drivers of the conformational switch and the engagement of the ASM_4_ vary depending on differences in amino acid sequences.

While Tat^Fin^ has two major differences from Tat^NL4-3^ (K53 to R53 and spacer H54 to Q54, respectively), it only differs by a single amino acid from HEXIM (R52 and K151, respectively). Like Tat^NL4-3^, R52 is responsible for remodeling pseudo-ASM_3_ (Fig. 3c and Supplementary Fig. 7a). However, while R53 in Tat^NL4-3^ flips over R52 and engages ASM_4_, the equivalent K53 stays in the spacer region between the ASM_1_/ASM_2_ and ASM_3_/ASM_4_ regions in a manner similar to HEXIM as evidenced by NOEs between the K53 Hβ protons with the U_68_ H5 proton, the K53 Hδ protons with the C_67_ and U_66_ H5 protons, and the K53 Hε protons with the C_67_ H5 and H6 protons, which position the amino side chain of K53 within hydrogen-bonding distance of the C_67_ backbone (Fig. 3c and Supplementary Fig. 7a).

As for HEXIM, ASM_4_ remains unoccupied upon binding Tat^Fin^ and the structure shows that the K51 amino side chain is positioned to hydrogen-bond with the U_63_ ribose ring in a stabilizing interaction (Fig. 3c). This is evidenced by NOEs of the K51 Hδ protons with the U_63_ H5 and H1′ protons and the K51 Hε protons with the U_63_ 2′ hydroxyl proton (Supplementary Fig. 7c). Furthermore, the N-terminal K50 exits near the apical loop, with NOEs observed of the K50 Hδ and Hε protons with the C_38_ and C_37_ H5, and H1′ protons position the amino side chain of K50 to the C_38_ phosphate backbone (Fig. 3c and Supplementary Fig. 7a).

Finally, in evaluating the structural consequences of the spacer substitution, we see that H54 and R55 in Tat^Fin^ remain near ASM_1_ and ASM_2_, similar to what is found in HEXIM. This is evidenced by NOEs of the H54 (H153 in HEXIM) Hβ protons with the C_35_, C_36_, and C_37_ H5 protons, placing H54 near ASM_2_, whereas the R55 (R154 in HEXIM) Hδ protons display NOEs with the A_34_ H1′ proton and the C_33_ H1’, H5, and H6 protons, positioning this spacer residue near ASM_1_ (Fig. 3d and Supplementary Figs. 6, 7). This is in contrast with the binding mode of Tat^NL4-3^ in which the intercalation of R53 into ASM_4_ drags both the Q54 and R55 spacer residues towards the apical ASMs.

The importance of the histidine H54 spacer is even more evident in the Tat^G^ strain where it represents the only difference from Tat^NL4-3^. This single difference changes the identity of the arginine that remodels pseudo-ASM_3_. In this ARM, the positioning of H54 near ASM_2_ precludes R53 from reaching ASM_4_ to accomplish the inverse intercalation seen in NL4-3 (Fig. 3d and Supplementary Fig. 7d, e). The interactions with the apical ASMs thus occur in a ladder-like manner where R53 intercalates into the remodeled ASM_3_ whereas R52 intercalates into ASM_4_ (Fig. 3c, d and Supplementary Fig. 7a, d, e). K51 makes the final stabilizing interaction with NOEs seen between the Hε protons and the U_63_ 2′ hydroxyl proton, indicating a hydrogen-bonding interaction between the K51 amino side chain and the U_63_ ribose ring (Fig. 3d and Supplementary Fig. 7f). Taken together, these studies show that arginine sandwich motifs provide mini domains that arginine-rich motifs of proteins can differentially interact with to achieve deep major groove binding into the stem of 7SK-SL1^apical-AGU^.

### HEXIM allows for increased conformational sampling of apical ASMs

**Fig. 4:**
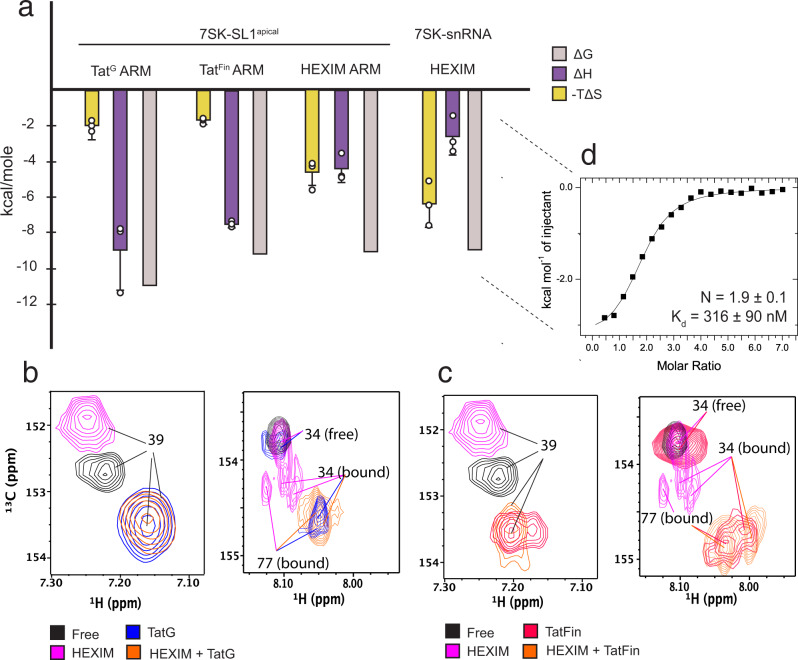
Comparative thermodynamic analyses and competition experiments between Tat and HEXIM. **a** Comparison of enthalpic and entropic contributions between Tat^G^, Tat^Fin^, and HEXIM^N-ARM^ in complex with 7SK-SL1^apical-AGU^, and full-length HEXIM in complex with 7SK snRNA. Entropy values were calculated using a T value of 298 K. The reversal in the entropic and enthalpic contribution for Tat ARM compared to HEXIM is evident with HEXIM having an entropically-driven binding profile. NMR competition titration analysis showing binding of 7SK by Tat^G^ (**b**) and Tat^Fin^ (**c**) concomitant with the total displacement of HEXIM^N-ARM^. Data are shown for the A_39_ (left) and A_34/77_ (right) h2-c2 correlations. The increase in Tat engagement of ASM_1_ for the HEXIM^N-ARM^-bound complex is evident by the lack of free-RNA populations for the A_34_ resonance in the competition experiment compared to binding to free 7SK. Furthermore, the destabilization of A_34_ in ASM_1_ by HEXIM is indicated by multiple bound states. Also shown for comparison is the complete engagement of A_39_ by all ARMs. **d** Representative ITC data for full-length HEXIM bound to 7SK snRNP demonstrating expected stoichiometry and specific binding. All reported values are for *n* = 3 replicates.

While titration of all four arginine-rich motifs stabilizes the majority of 7SK-SL1^apical-AGU^ into one predominant configuration, the HEXIM ARM is an outlier wherein binding causes ASM_1_ and ASM_4_ to become destabilized and exhibit multiple conformations (Fig. 4a, b and Supplementary Fig. 4). In such conformations, the NOEs between the imino protons of U_63_ and U_44_ disappear, indicating the disruption of the U_63_:U_44_-A_65_ triple and loss of ASM_4_ (Supplementary Fig. 8c). The destabilization of this region is also indicated by the line-broadening of K150, which interacts with U_63_ in the folded configuration (Supplementary Fig. 6e).

The destabilization of 7SK-SL1^apical-AGU^ only by HEXIM is further evident when comparing the thermodynamic profiles between Tat and HEXIM. The binding of Tat^Fin^ and Tat^G^ strains is enthalpically driven (ΔH = −7.5 ± 0.2 and −8.9 ± 2.2 kcal mol^−1^, respectively; Fig. 4c) with a modest entropic contribution (−TΔS = −1.7 ± 0.3 and −2 ± 0.8 kcal mol^−1^, respectively; Fig. 4c). On the other hand, HEXIM binding is entropically enhanced by ~2.5-fold over both Tat strains (−TΔS = −4.6 ± 0.8 kcal mol^−1^, ΔH = −4.4 ± 0.7 kcal mol^−1^; Fig. 4c). The Brillet et al. study performed in 0.5 M salt saw an unfavorable entropic contribution for Tat and a negligible entropic for HEXIM binding, underscoring the importance of maintaining native ASM folding for a mechanistic understanding of this biological process^52^. Nevertheless, the overall observation that HEXIM binding is comparatively more entropic than Tat agrees with our results^52^.

To evaluate the implication of HEXIM’s ability to locally destabilize ASM_1_ and ASM_4_ in the context of its displacement required for transcriptional regulation, we compared Tat^Fin^ and Tat^G^ ARM binding to 7SK-SL1^apical^ both free and in the presence of HEXIM^N-ARM^. Due to the modest differences in binding energetics between the different ARMs, competition experiments using ITC were not tractable. A 1:1 titration of both Tat^G^ and Tat^Fin^ into 7SK in the NMR shows the ability to completely engage ASM_2_ and ASM_3_, while a significant fraction of ASM_1_ and ASM_4_ shows the presence of free configurations, indicating reduced access for the termini. However, upon titration of both Tats into the HEXIM-bound 7SK complex, we observe not only complete engagement of all ASMs but also a total displacement of HEXIM (Fig. 4a, b). This is especially striking given that the binding affinities of Tat^Fin^ and HEXIM for free 7SK are equivalent. Taken together, these data indicate that Tat can better engage 7SK that is destabilized by HEXIM at the outer ASMs. Finally, ITC data of full-length HEXIM bound to full-length 7SK snRNA (*N* = 1.9 ± 0.1; Fig. 4d) show that an entropy-driven interaction is maintained and, in fact, is even more pronounced (−TΔS = −6.4 ± 1.3 kcal mol^−1^, ΔH = −2.6 ± 1 kcal mol^-^1; Fig. 4c), suggesting that HEXIM binding may globally increase the conformational space sampled by the 7SK snRNP complex. These studies suggest that destabilization by HEXIM may play an important role in how transcription factors access 7SK for pTEFb capture.

## Discussion

The 7SK snRNP represents a central biomolecule that a wide range of transcriptional factors needs to interact with to access pTEFb to control transcriptional elongation. In particular, pTEFb extraction by HIV Tat from this complex requires manipulating the interaction between the 7SK snRNA and the HEXIM adapter protein. In this study, we solved the structures of the RNA binding domains of HEXIM and Tat bound to 7SK and gained several insights into their functional significance, including the malleability of 7SK, the local destabilization by HEXIM, and the specific sequence variations of Tat.

The structures show that both HEXIM and Tat directly bind the stem of 7SK-SL1^apical^ through intercalation of arginine-rich motifs into an entire helical turn of the major groove. This is unusual as RNA major grooves are deep and narrow, making them generally inaccessible for protein binding. The architecture of the four sandwich motifs in 7SK allows for transcriptional regulators to differentially utilize their ARMs. On the one hand, the tandem preformed ASMs, ASM_1_ and ASM_2_, remain unchanged from their free configuration upon encountering the C-terminal arginines of Tat and HEXIM. On the other hand, the apical pseudo-symmetrical ASMs, pseudo-ASM_3_, and ASM_4_, reconfigure depending on their binding partners. The structures show that the ASM_3_ region can adopt at least three different base pair interactions: a reverse Hoogsteen in the free state, a cis-Hoogsteen/sugar interaction upon HEXIM binding, and a Watson–Crick base pair upon Tat binding. The cis-Hoogsteen/sugar interaction is especially significant because it allows HEXIM to enter the major groove despite the lack of arginines in the N-terminus. Similarly, while ASM_4_ retains its preformed configuration found in the free state upon Tat binding, it can be destabilized in the presence of HEXIM and adopt multiple flexible states. Taken together, these studies show that 7SK is adaptable in its ASM architecture, which can be modulated upon encountering different transcription factors.

Comparative analyses of HEXIM and Tat provide insights into how both positive and negative regulators can manipulate 7SK to carry out their transcription roles. Our studies implicate HEXIM as potentially having a dual structural role. On the one hand, it can bind with high affinity to the apical portion of 7SK-stemloop-1, and on the other hand, it simultaneously causes local destabilization of this region, enhancing the binding of a positive regulator such as Tat. In comparison to Tat, the thermodynamic profile and solution-state characteristics of HEXIM binding show an entropy-driven mode of interaction that is particularly attributed to the destabilization of ASM_1_ and ASM_4_ regions, indicating a mechanism in line with conformational selection. Indeed, mutational studies have shown that deletion of U_63_ significantly reduces HEXIM binding^37,43^. This expansion in the dynamic state of 7SK surrounding the ASM_1_ and ASM_4_ region is also supported both by in vivo SHAPE analysis where U_63_ and C_75_ become ultra-reactive upon HEXIM:pTEFb binding^53^. Such increased conformational sampling was also demonstrated by structural and molecular dynamics modeling^45,52–57^. Furthermore, we show that Tat capitalizes on this increased dynamic state, binding to more motifs with greater affinity to the HEXIM-bound complex than to free 7SK. While the use of a HEXIM-displacement mechanism for pTEFb capture by binding to 7SK-SL1 has yet to be discovered for cellular factors, the destabilization-driven preparation of 7SK snRNP may potentially be a general feature exploited by specialized transcriptional factors.

Comparative analysis of HEXIM and Tat also sheds light on the sequence requirements of ARMs for 7SK binding. While N-terminal lysines of HEXIM allow for destabilization of ASM_4_, the anchoring required to enter the major groove can only be provided by the stacking of C-terminal arginines within ASM_1_ and ASM_2_. Indeed, the importance of these C-terminal arginines for HEXIM binding is supported by their nearly complete conservation across metazoan species^58^. Conversely, the equivalent arginines in HIV-1 Tat occur as a consecutive pair only in ~50% of reported strains, albeit with the strong requirement of at least one arginine. The structures show that these variations may be possible due to the anchoring provided by the arginines that intercalate into the apical ASMs. Nevertheless, when two arginines are present in Tat, the interactions with the tandem ASMs mirror HEXIM.

Furthermore, differences in N-terminal and spacer ARM residues orchestrate the structural modulations of the apical ASMs. To accommodate the continuation of the HEXIM ARM chain from the ASM_1_/ASM_2_ to the ASM_3_/ASM_4_ region required for U_63_ destabilization, K152 induces the reconfiguration of pseudo-ASM_3_ from a reverse Hoogsteen to a cis-Hoogsteen/sugar interaction. In all variations of N-terminal Tat sequences studied, binding is concomitant with the rearrangement of pseudo-ASM_3_ into a canonical ASM_3_ through the intercalation of an arginine.

The structures also provide insights into specific sequence variations that occur in the highly conserved Tat ARM to displace HEXIM. When two arginines are available in the N-terminal residues, both are involved in arginine sandwich interactions, providing a twofold increase in affinity; however, either R52 (Tat^NL4-3^) or R53 (Tat^G^) can act as the remodeler. This can be explained by the presence of either glutamine or histidine spacer, respectively, which is the only amino acid difference between the two strains. As glutamine (75%) and histidine (15%) make up most of the sequence variation in this spacer, the structures show that these two spacer residues drive the differential positioning of the arginine remodeler. In the Tat^Fin^ strain, which has a histidine spacer, it is the R52 that acts as a remodeler. In this case, the R53K substitution provides the stabilizing interactions to reposition the single R52 arginine near pseudo-ASM_3_.

It is also interesting to compare the mode of binding of Tat^Fin^ to HEXIM. First, the single residue difference (R52 vs K151) provides Tat^Fin^ with the additional ASM intercalation required for displacement. Thus, Tat has evolved specific sequence variations that allow for the reconfiguration of pseudo-ASM_3_, even in cases where there is only a single variation from HEXIM. Second, despite both ARMs having lysines positioned near ASM_4_, only HEXIM leads to local destabilization. Our studies, therefore, provide HEXIM as an example of a negative regulator that primes its own displacement by locally destabilizing 7SK. Overall, these studies have broader implications for 7SK snRNP-mediated regulation. Given that the destabilization-driven displacement is a robust mechanism, it is possible that other yet-to-be-identified cellular and viral transcriptional regulators recruit pTEFb through direct intercalation of ARMs into 7SK-SL1^apical^. Furthermore, as a destabilized state of 7SK snRNP is what is presented to all transcriptional regulators, the mechanisms necessary to extract pTEFb may converge on capitalizing on this conformational heterogeneity.

## Methods

### RNA sample preparation

RNA samples used for biophysical experiments were synthesized by in vitro transcription using T7 RNA polymerase with either plasmid DNA or with synthetic DNA templates containing 2′-O-methylated (Integrated DNA Technologies) containing the T7 promoter and the desired sequences. Plasmid DNA for 7SK-SL1^Full-WT^ and 7SK-SL1^Full-AGU^ containing the T7 promoter, insert, and SmaI recognition sequence were cloned by Genscript in between the EcoRI and BamHI restriction sites of a puc19 vector. Plasmid DNA was prepared for in vitro transcription from a 5 mL overnight culture of NEB 5α Competent *E.coli* (C29871) transformed with the plasmid using Qiaprep Spin Miniprep Kit (Qiagen 27104). 10 μL of purified DNA were combined with 25 μL of 2′-O-methylated reverse primer at 100 μM (5′-mGmGAGCGGTGAGG GAGGAAG-3′ where m indicates 2′ O-methylated nucleotides), 25 μL of forward primer at 100 μM (5′-GACAAGCCCGTCAGGG-3′), 2.44 mL of water, and two tubes of EconoTaq PLUS 2X Master Mix (Lucigen 30035-2). The 5 mL mixture was then aliquoted into 50 μL increments in a 96-well PCR plate and the templates for in vitro transcription reactions were amplified using the following PCR protocol: 95 °C for 5 min, 34 cycles of (95 °C for 30 s, 50 °C for 1 min, and 68 °C for 90 s), and 68 °C for 5 min. After PCR amplification, reactions were pooled into 5 mL volume in a 50 mL Falcon tube and 0.5 mL of 3 M sodium acetate, pH 5 and 32 mL of 100% ethanol were added to the mixture and chilled at −80 °C for at least 30 min before spinning down at 9000×*g* for 10 min at 4 °C. The ethanol was decanted, and the pellet was left to dry overnight before in vitro transcription use. Template preparation for 7SK-SL1^apical-AGU^ using 2′-O-methylated reverse primers in order to suppress the heterogeneity at the 3′ end of the transcripts involved combining 15 mL of both forward (5′-TAATACGACTCACTA TAGGGATCTGTCACCCCATTGATCGCCAGTGGCTGATCTGGCTGGCTAGGCGGGTCCC-3′) and reverse (5′-mGmGGACCCGCCTAGCCAGCCAGATCAGCCACTGGC GATCAATGGGGTGACAGATCCCTATAGTGAGTCGTATTA-3′ where m indicates 2′ O-methylated nucleotide) primers at 1 mM stock solution with 470 mL of water^59^. The mixture was heated at 95 °C for 5 min and cooled at room temperature for 30 min before assembling the in vitro transcription reaction. Samples were either unlabeled or residue-specifically labeled with ^13^C/^15^N- or ^2^H (Cambridge Isotope Laboratories, Inc.). After transcription, RNA samples were heat denatured and purified by using urea-denaturing polyacrylamide gels. The same in vitro transcription reaction protocol was done for 7SK-SL1^apicalΔASM^ using a forward (5′-TAATACG ACTCACTATAGGGATCTGTCACCCCAGATCGCCAGTGGCGATCTGGGGAGGCGGGTCCC-3′) and reverse (5′-mGmGGACCCGCCTCCCCAGATCGCCACTGGCGATCT GGGGTGACAGATCCCTATAGTGAGTCGTATTA-3′ where m indicates 2′ O-methylated nucleotide).

### HEXIM ARM and Tat ARM peptide preparation

Unlabeled HEXIM^N-ARM^ (GISYGRQLGKKKHRRRAHQ), Tat^Fin^ ARM (GISYGRKKRKHRRRAHQ), and Tat^G^ ARM (GISYGRKKRRHRRRAHQ) peptides were purchased from Tufts University Core Facility at a 0.1 mmol scale. Tat adapters were placed around the HEXIM^N-ARM^ sequence to prevent non-physiological aggregation in solution-state NMR studies. HEXIM^N-ARM^ peptides containing selective ^13^C/^15^N_-_labeled residues, underlined, (GISYGRQLGKKKHRRRAHQ and GISYGRQLGKKKHRRRAHQ) were purchased from New England Peptide.

### Full-length HEXIM1 preparation

Synthetic DNA encoding HEXIM1 (2-359) was cloned into a bacterial pMCSG7 expression vector^59^ encoding an N-terminal tobacco etch virus (TEV) protease-cleavable His_6_ tag and was expressed in *E. coli* BL21 AI cells in an overnight culture at 20 °C. Cells were lysed by sonication in buffer containing 50 mM Tris pH 8.0, 500 mM NaCl, 0.1% β-mercaptoethanol, 50 mM (NH_4_)_2_SO_4_ and protease inhibitor aprotinin and leupeptin. His_6_-HEXIM1 was purified from the cleared cell lysate using Ni-NTA resin (Qiagen) and the His_6_ tag was cleaved with TEV protease. The HEXIM was run over a second Ni-NTA column, followed by anion exchange on a 5 mL HiTrap Q HP column (Cytiva) and gel filtration on a Superdex 200 16/60 column (Cytiva) in a final buffer containing 25 mM HEPES pH 7.5, 200 mM NaCl, 5% glycerol, 1 mM TCEP. HEXIM was flash frozen in liquid nitrogen and stored at −80 °C.

### Isothermal titration calorimetry

Binding constants for the interactions of 7SK-SL1^apical-AGU^ with the HEXIM^N-ARM^ and Tat^Fin^ and Tat^G^ ARMs and full-length HEXIM1 with 7SK-SL1^apical-AGU^, 7SK-SL1^Full-WT^, and 7SK-SL1^Full-AGU^ were measured using an ITC-200 microcalorimeter (MicroCal). 68 μM HEXIM^N-ARM^ peptide was titrated into 5 μM solutions of 7SK-SL1^apical-AGU^ or 7SK-SL1^apicalΔASM^ in 10 mM sodium phosphate, 70 mM NaCl, 0.1 mM EDTA, pH 5.2 at 25 °C. Titrations of Tat ARMs into 7SK-SL1^apical-AGU^ or 7SK-SL1^apicalΔASM^ were also performed in the same buffer conditions as the HEXIM^N-ARM^ titration, although the Tat ARM concentration was at 2.5 μM and the 7SK-SL1^apical-AGU^ concentration was at 45 μM. Titrations with full-length HEXIM1 were done at 100 μM of HEXIM1 into 3 μM of either 7SK-SL1^apical-AGU^, 7SK-SL1^Full-WT^, and 7SK-SL1^Full-AGU^ in a buffer of 25 mM HEPES pH 7.5, 200 mM NaCl, 5% glycerol, and 1 mM TCEP. Titration curves were analyzed using ORIGIN (OriginLab) and all thermodynamic parameters are reported with *n* = 3 experiments.

### Small-angle X-ray scattering

SAXS data for the 7SK-SL1^apical-AGU^:HEXIM^N-ARM^, 7SK-SL1^apical-AGU^: Tat^Fin^ ARM, and 7SK-SL1^apical-AGU^:Tat^G^ ARM complexes were obtained at SIBYLS beamline of Advanced Light Source at Lawrence Berkeley National Laboratory. Measurements were performed in a buffer containing 10 mM sodium phosphate, 70 mM NaCl, 0.1 mM EDTA, pH 5.2, and the background scattering was subtracted from the sample scattering to obtain the scattering intensity from the solute molecules. Data from three different concentrations (50, 75, and 100 μM) were compared with scattering intensities at *q* = 0 Å^−1^ [I(0)], as determined by Guinier analysis, to detect possible interparticle interactions. Data were analyzed by using ScÅtter software, and the presented DAMAVER envelope structures were reconstructed by using DAMMIF/DAMMIN software from 23 independent DAMMIF runs. Chi-squared values of SAXS profiles were analyzed on FoXS^60,61^.

### NMR data acquisition, resonance assignment, and structural calculations

For NMR experiments, the Tat ARM/HEXIM^N-ARM^:7SK-SL1^apical-AGU^ complexes were dissolved in a buffer containing 10 mM potassium phosphate, 70 mM NaCl, and 0.1 mM EDTA, pH 5.2, whereas the full-length HEXIM1:7SK-SL1^apical^ complex was in a buffer with 25 mM HEPES pH 7.5, 200 mM NaCl, 5% ^2^H-glycerol, and 1 mM TCEP. All NMR experiments were acquired by using Bruker 700 or 800 MHz instruments equipped with cryogenic probes. Spectra for observing non-exchangeable protons were collected at 298 K in 99.96% D_2_O, whereas those for exchangeable protons were at 283 K and 298 K in 10% D_2_O. For NOESY experiments, mixing times were set to 200 ms. To help unambiguously assign the intermolecular NOEs of the HEXIM^N-ARM^ with 7SK-SL1^apical-AGU^, we used both specifically protonated GA, AC, and GU samples of 7SK-SL1^apical-AGU^ and two HEXIM^N-ARM^ peptides synthesized by with different combinations of ^13^C/^15^N-labeled amino acids. Samples of the 7SK-SL1^apical-AGU^:HEXIM^N-ARM,^ the 7SK-SL1^apical-AGU^:Tat^Fin^ ARM, and 7SK-SL1^apical-AGU^:Tat^G^ ARM were prepared at 1:0.9 equivalents, whereas the full-length HEXIM1:7SK-SL1^apical-AGU^ complex was prepared at 1:0.3 equivalents to avoid any nonspecific binding or aggregation of the protein to the RNA. Assignments for non-exchangeable ^1^H, ^13^C, ^15^N signals of 7SK-SL1^apical-AGU^ in complex with HEXIM^N-ARM^ and Tat ARMs were obtained by analyzing two-dimensional ^1^H-^1^H NOESY recorded with non-labeled samples and two-dimensional ^13^C-HMQC and ^15^N-HSQC and three-dimensional ^13^C-edited HMQC-NOESY spectra for labeled samples.

Initial structural models were generated using manually assigned restraints in CYANA, where upper-limit distance restraints of 2.7, 3.3, and 5.0 A were employed for direct NOE cross-peaks of a strong, medium, and weak intensities, respectively^62^. However, for cross-peaks pairs associated with the intra-residue H8/6 to H2′ and H3′, upper distance limits of 4.2 and 3.2 Å were employed for NOEs of medium and strong intensity, respectively. To prevent the generation of structures with collapsed major grooves, cross-helix P–P distance restraints (with 20% weighting coefficient) were employed for A-form helical segments. Standard torsion angle restraints were used for regions of A-helical geometry, allowing for ±50° deviations from ideality (α = −62°, β = 180°, γ = 48°, δ = 83°,ɛ = −152°,ζ = −73°)^63^. Standard hydrogen-bonding restraints with approximately linear NH–N and NH–O bond distances of 1.85 ± 0.05 Å and N–N and N–O bond distances of 3.00 ± 0.05 Å, and two lower-limit restraints per base pair (G–C base pairs: G-C4 to C-C6 ≥ 8.3 Å and G-N9 to C-H6 ≥ 10.75 Å; A–U base pairs: A-C4 to U-C6 ≥ 8.3 Å and A-N9 to U-H6 ≥ 10.75 Å) were employed in order to weakly enforce base-pair planarity (20% weighting coefficient).

The CYANA structure with the lowest target function was used as the initial model for structure calculations Xplor-NIH to incorporate electrostatic constraints. First, structures were calculated using annealing from 2000 °C to 25 °C in steps of 12.5 °C. Standard energy potential terms for bonds, angles, torsion angles, van der Waals interactions, and interatomic repulsions were included. The statistical backbone H-bond potential was utilized for protein residues. Energy potentials for NOEs, hydrogen bonds, and planarity were incorporated with restraints derived from NMR data. All restraints used in CYANA were included except for phosphate-phosphate distances. The structures were sorted by energy using bond, angle, dihedral, and NOE energy potential terms, and the ten percent of the structures with the lowest sort energy were further minimized with SAXS terms to incorporate orientation restraints. For this step, minimization started at 1500 °C to 25 °C in steps of 12.5 °C. The lowest ten percent of these were deposited in the RCSB databank.

### Reporting summary

Further information on research design is available in the Nature Research Reporting Summary linked to this article.

## Supplementary information

Supplementary Material

Reporting Summary

## Data Availability

Atomic coordinates have been deposited in the Protein Data Bank under accession codes PDB 7T1N (7SK-SL1^apical-AGU^:HEXIM^N-ARM^), PDB 7T1P (7SK-SL1^apical-AGU^:Tat^Fin^), and PDB 7T1O (7SK-SL1^apical-AGU^:Tat^G^). Chemical shifts have been deposited in the Biological Magnetic Resonance Data Bank under accession codes 30971 (7SK-SL1^apical-AGU^:HEXIM^N-ARM^), 30973 (7SK-SL1^apical-AGU^:Tat^Fin^), and 30972 (7SK-SL1^apical-AGU^:Tat^G^). SAXS data were submitted to and validated by SASBDB (https://www.sasbdb.org/)^64^ under accession codes SASDME9 (7SK-SL1^apical-AGU^:HEXIM^N-ARM^), SASDMF9 (7SK-SL1^apical-AGU^:Tat^Fin^), and SASDMD9 (7SK-SL1^apical-AGU^:Tat^G^). Source data are available through Dryad (10.5061/dryad.12jm63z17) and upon reasonable request from the corresponding author.
