# Supplementary material for: A structure-based mechanism for displacement of the HEXIM adapter from 7SK small nuclear RNA
Source: Commun Biol. 2022 Aug 15;5:819. doi: 10.1038/s42003-022-03734-w (PMC9378691; doi:10.1038/s42003-022-03734-w)
Supplement: Supplementary file 1 — Supplementary Material [file 42003_2022_3734_MOESM1_ESM.pdf]

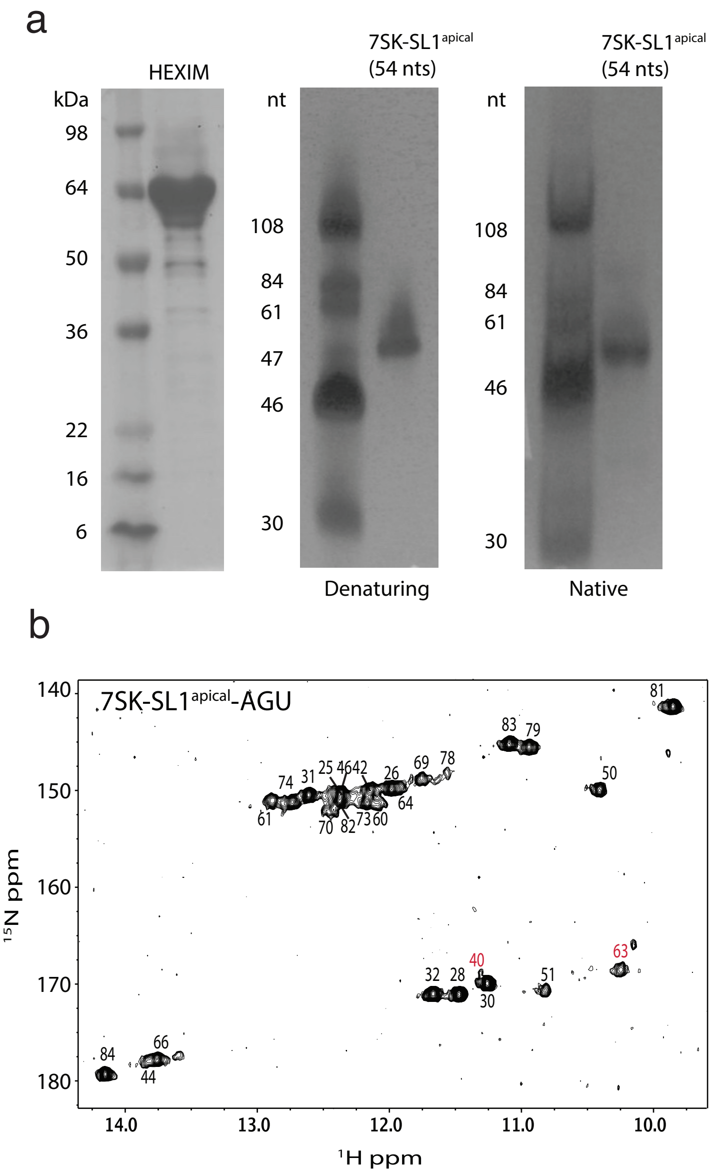

Supplementary Figure 1: HEXIM1 and 7SK-SL1$^{apical\text{-}AGU}$ purification

(**a**) Coomassie stained SDS-PAGE showing HEXIM after purification (left). HEXIM has a molecular weight of 40.4 kDa, but has been shown to run with an apparent molecular weight closer to 67 kDa[35]. Denaturing (middle) and native (right) gels of the 56 nucleotide 7SK-SL1$^{apical\text{-}AGU}$ purified RNA product. **(b)** Two-dimensional ^{1}H-^{15}N HSQC spectra for 7SK-SL1^{apical} with an AGU triloop (top) and a native loop (bottom) showing that all major motifs in the apical stem are unaffected by the change in the loop. Expected chemical shifts match the HSQC spectra of 7SK-SL1$^{apical\text{-}WT}$ is from previous publication[45].

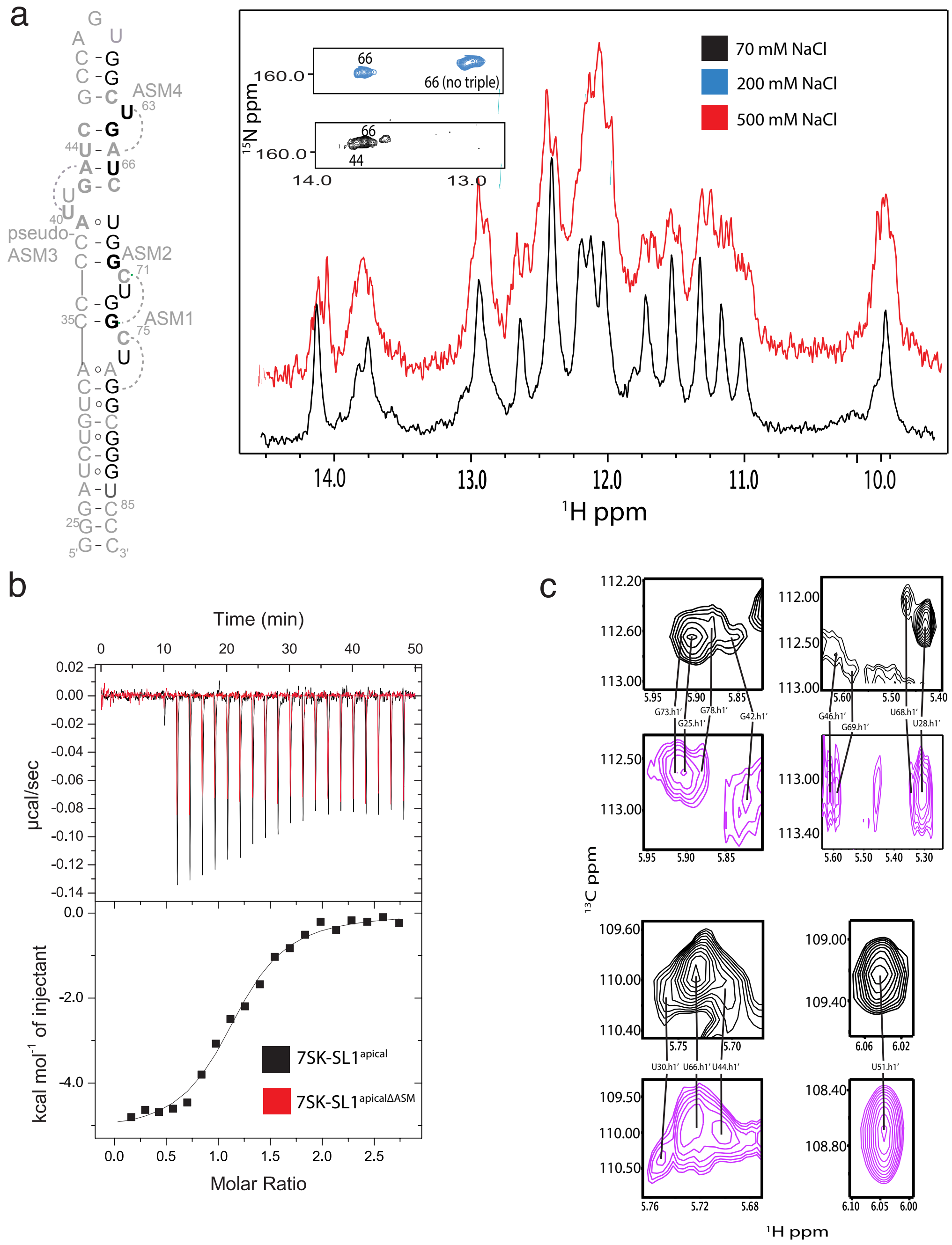

Supplementary Figure 2: Determination of experimental conditions for characterizing the minimal 7SK-SL1$^{apical-AGU}$:HEXIM N-ARM interaction.

(**a**) Two-dimensional ^{1}H-^{15}N HSQC spectra of 7SK-SL1$^{apical-AGU}$ where residues labeled in black were specifically labeled under high salt conditions. The emergence of a non-tripled based U_{66} imino at ~13.1ppm (inset), the disappearance of the U_{63} imino, and the upfield shift of the G_{79} imino to ~10.2ppm shows the dissolution of the ASMs under high salt conditions. Comparison of 1D ^{1}H experiments of 7SK-SL1$^{apical-GAGA}$ in 500mM NaCl (blue)

used in a previous study[52] and 70mM NaCl (red). High salt leads to the significant broadening of peaks and the emergence of new imino resonances not seen near physiological conditions. **(b)** Representative ITC baseline subtraction for generation of ITC curves. Heat shown in red represent an ITC experiment of the HEXIM$^{\text{N-ARM}}$ peptide titration into just a 7SK-SL1$^{\text{apical-AGU}}$ construct lacking bulge pyrimidine residues with the AGU triloop. This baseline subtraction protocol was applied to all peptide ITC experiments. **(c)** Two-dimensional ^{1}H-^{13}C HMQC spectra of GU-labeled 7SK-SL1$^{\text{apical-AGU}}$ binding to 0.3 equivalents of full-length HEXIM1 (black) and 0.9 equivalents of HEXIM N-ARM (magenta). While there are slight differences in both carbon and proton chemical shifts due to the salt concentration of the buffers, the HEXIM N-ARM makes the same interactions with 7SK-SL1$^{\text{apical-AGU}}$ as the full-length HEXIM1.

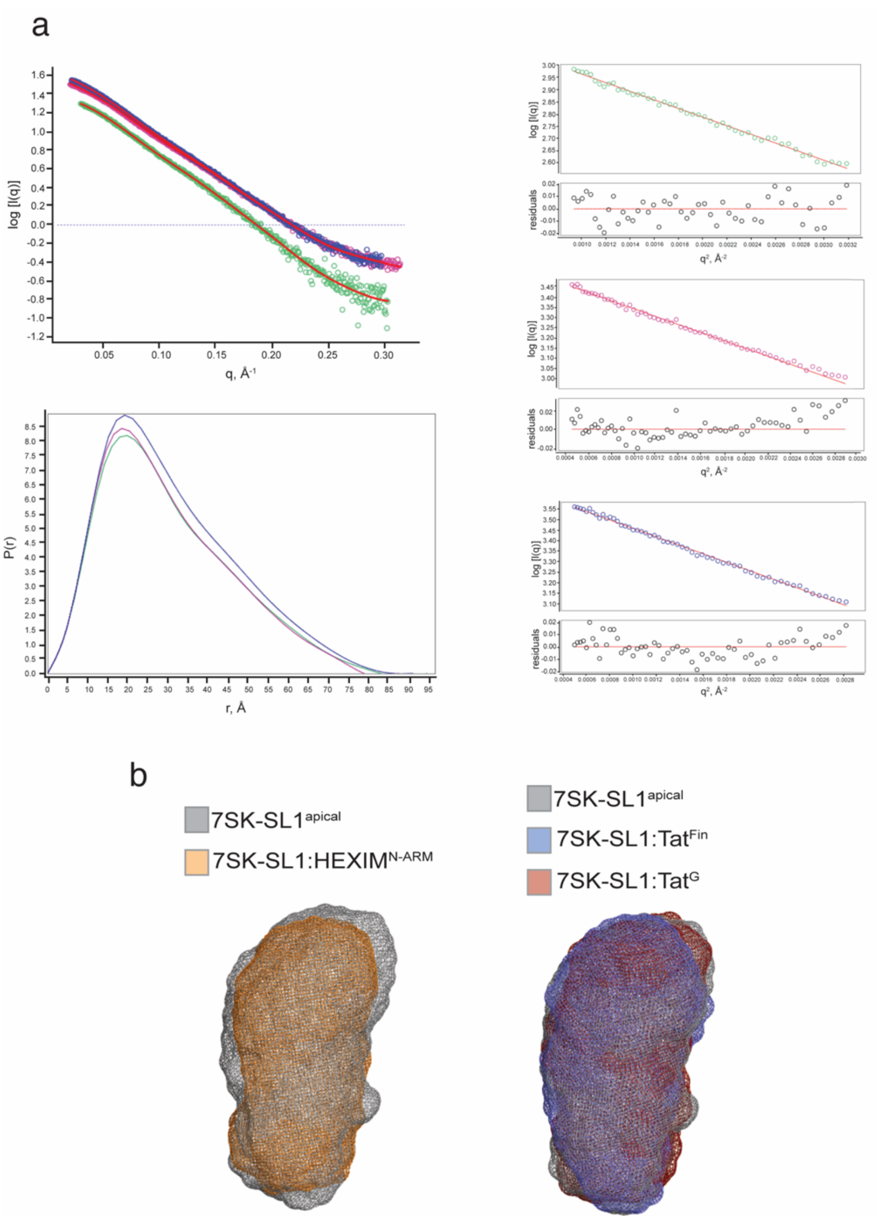

Supplementary Figure 3: SAXS analysis of 7SK-SL1$^{\text{apical-AGU}}$ free and in complex with HEXIM and Tat ARMs.

(**a**) Intensity (top left), pairwise distance distribution function (bottom left), and Gunier plots (right) for 7SK-SL1$^{\text{apical-AGU}}$ in complex with HEXIM$^{\text{N-ARM}}$ (magenta), Tat^{Fin} (blue), and Tat^{G} (green) ARMs. I is scattering intensity and q is proportional to the scattering angle ($q = 4\sin\theta/\lambda$ where 2θ is the angle between the incident x-ray beam and the detector, and λ is the x-ray wavelength in Å). **(b)** Overlay of reconstructed ab initio SAXS envelopes of free 7SK-SL1$^{\text{apical-AGU}}$ (gray) and in complex with HEXIM$^{\text{N-ARM}}$ (orange), Tat^{Fin} (blue), and Tat^{G} (red) ARMs, demonstrating the lack of global rearrangement of the RNA. Chi-squared values from FoXS analysis are 1.04 (7SK-SL1$^{\text{apical-AGU}}$), 1.68 (7SK-SL1$^{\text{apical-AGU}}$:HEXIM$^{\text{N-ARM}}$), 3.52 (7SK-SL1$^{\text{apical-AGU}}$:Tat^{Fin}), and 1.50(7SK-SL1$^{\text{apical-AGU}}$:Tat^{G}).

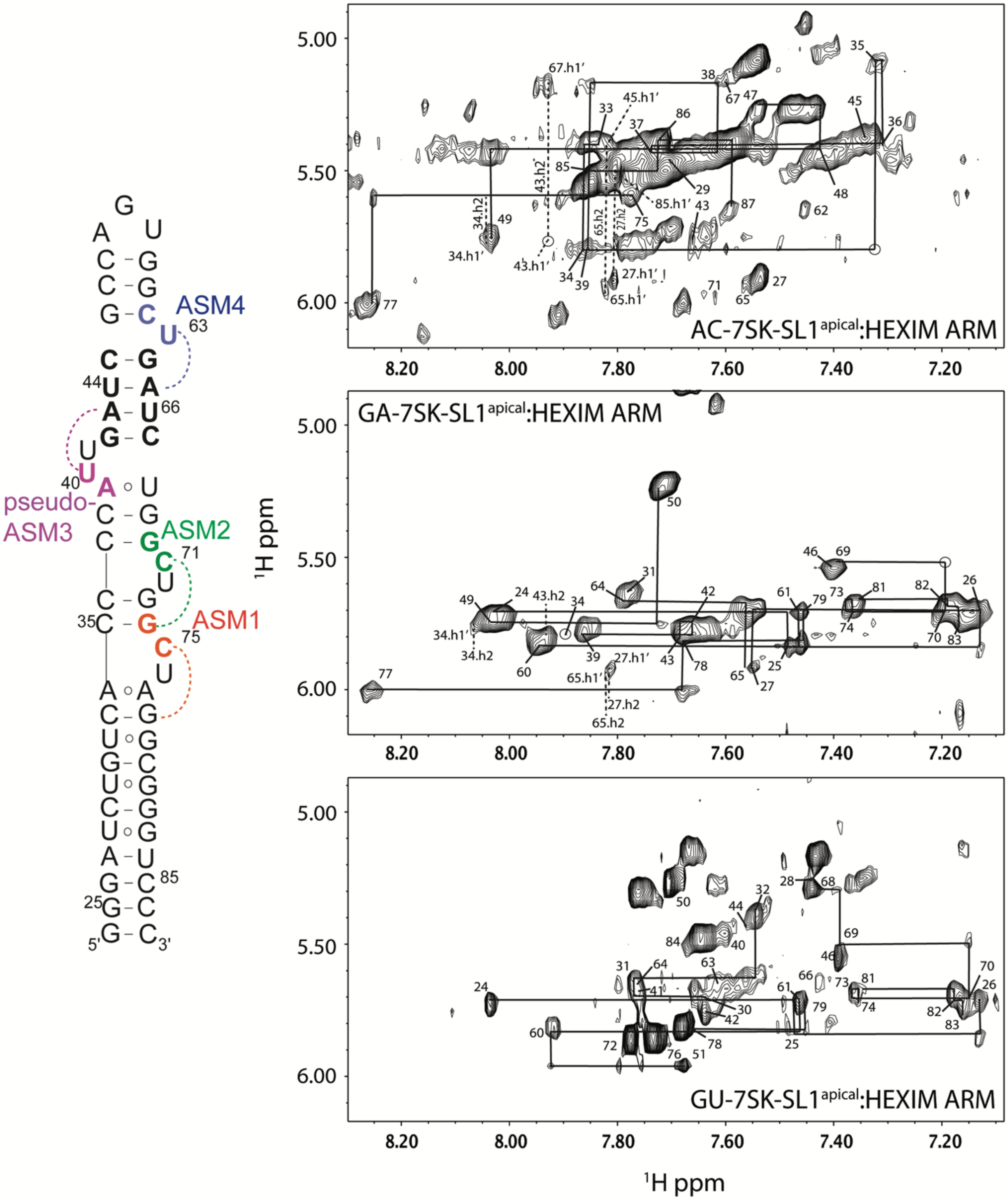

Supplementary Figure 4: Non-exchangeable RNA proton assignments of the HEXIM-N-ARM: 7SK-SL1$^{\text{apical-AGU}}$ complex.

Portion of two-dimensional ^{1}H–^{1}H NOESY spectra of the HEXIM-N-ARM:7SK-SL1$^{\text{apical-AGU}}$ interaction using AC (top), GU (middle), and GA (lower) protonated samples with the other nucleotides being deuterated.

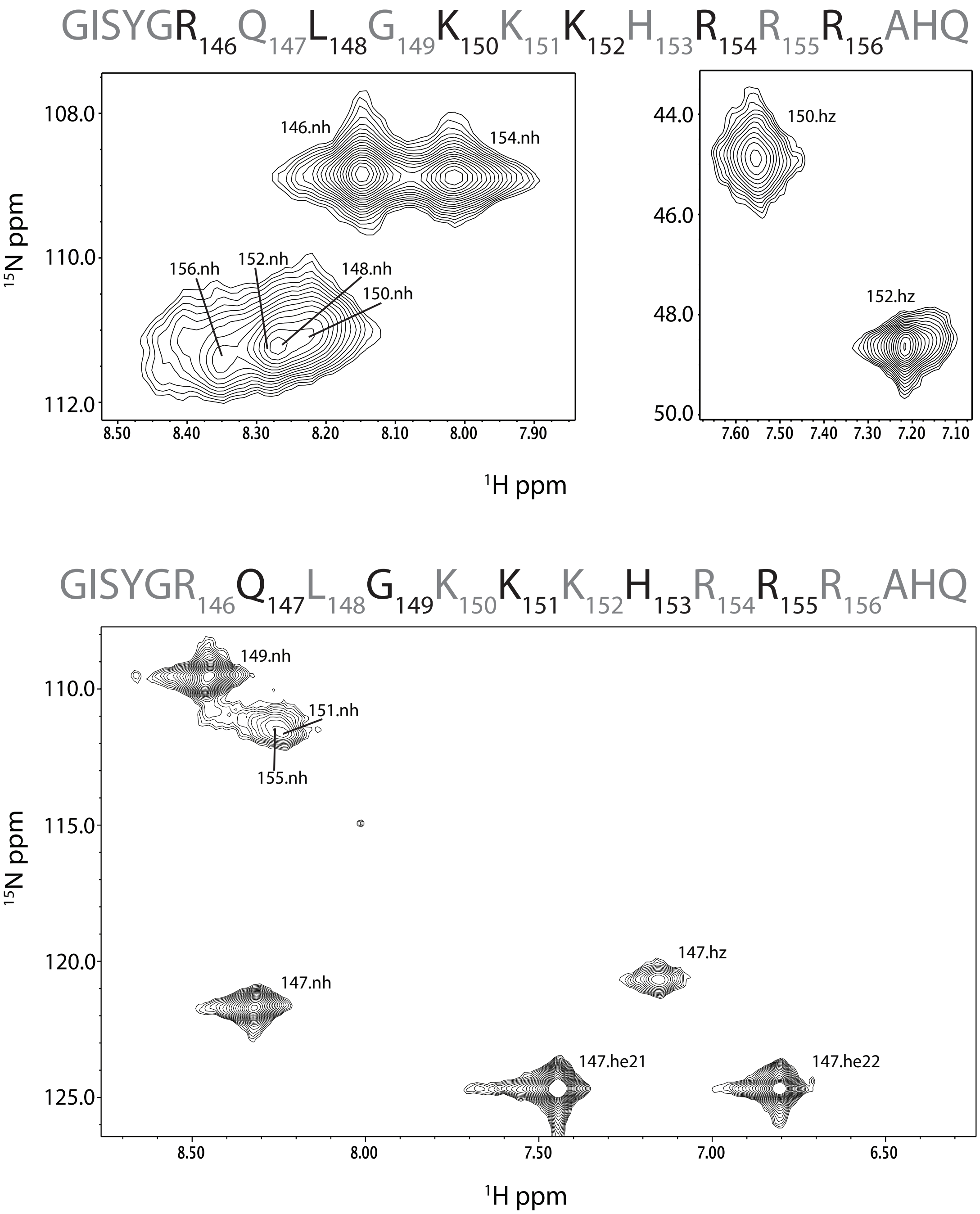

Supplementary Figure 5. Assignments of amino acid backbone and sidechain protons of the HEXIM N-ARM:7SK-SL1$^{apical\text{-}AGU}$ interaction.

HSQC spectra of the two selectively $^{13}C/^{15}N$- labeled HEXIM N-ARMs in complex with 7SK-SL1$^{apical\text{-}AGU}$. Bolded residues in the sequences are the amino acids that have been selectively labeled. All backbone amide protons and the side chain amino protons were unambiguously assigned with the help of this selective labeling strategy.

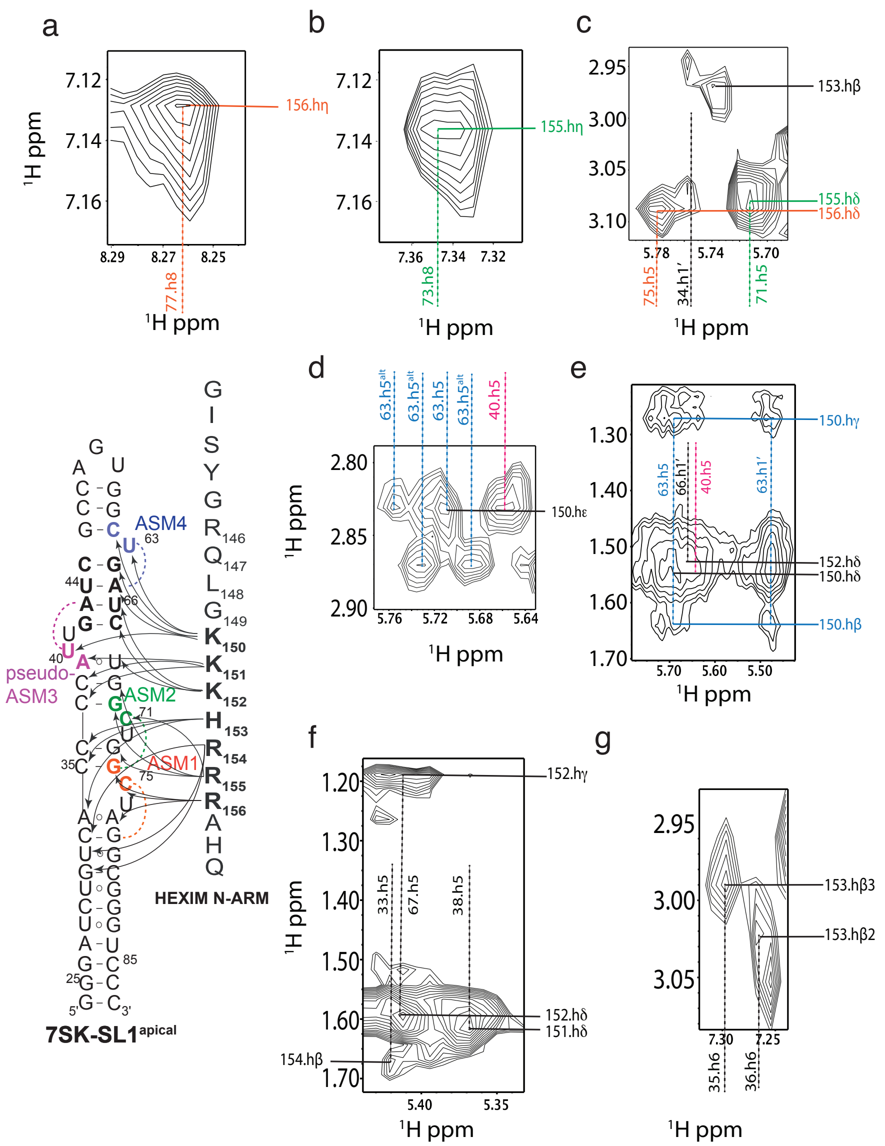

Supplementary Figure 6. Assignments of the interactions between the HEXIM N-ARM and 7SK-SL1$^{apical-AGU}$: Secondary structure of the 7SK-SL1$^{apical-AGU}$ showing connectivities to the HEXIM^{N-ARM}. NOEs between: **(a)** A_{77} H8 proton and R156 guanidinium protons indicate R156 docks into the preformed ASM_1; **(b)** G_{74} and G_{73} with the guanidinium protons of R156 and R155, respectively, and **(c)** 156 and 155 Hδ protons and H5 protons of the C_{75} and C_{71}^+ caps support entry of these arginines into ASM_1 and ASM_2; **(d)** K150 Hε protons and the U_{63} and U_{40} caps position K150 within hydrogen-bonding distance to the O4 of both residues. **(e)** K150 interacts with both the U_{63} and U_{40} caps of pseudo-ASM_3 and ASM_4 whereas interactions between the U_{66} H1′ protons with the K152 Hδ protons positions K152 as a spacer residue. **(f)** Interactions of K152 and K151 with the C_{67} and C_{38} H5 protons, respectively, and NOEs between the R154 Hβ proton with C_{33} determine the relative position of this spacer residue. **(g)** Positioning of the H153 Hβ protons with the H6 protons of C_{35} and C_{36}.

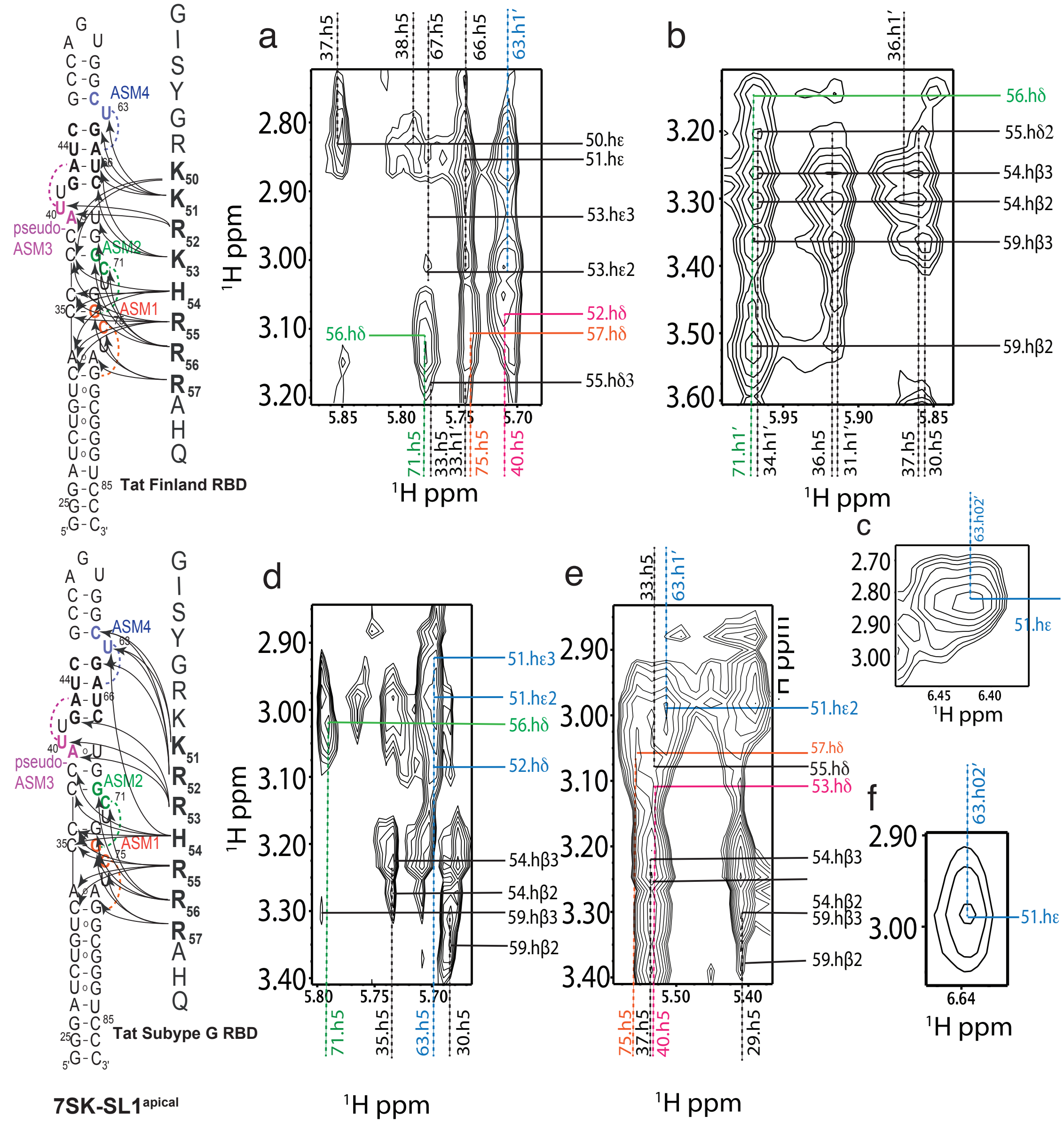

Supplementary Figure 7. Interactions between 7SK-SL1$^{apical-AGU}$ and Tat^{Fin} and Tat^{G} RBDs: Secondary structure of the 7SK-SL1$^{apical-AGU}$ showing connectivities to the RBDs. NOEs between: **(a)** R57, R56, and R52 Hδ of Tat^{Fin} protons and the C_{75}, C_{71}^{+}, and U_{40} caps position then into ASM_1, ASM_2, and remodeled ASM_3; K53 Hε and C_{67} and U_{66}, and R55 Hδ and C_{33} position these spacer residues in-between the lower and apical ASMs; **(b)** K51 Hε and U_{63} and U_{66}, and K50 Hε and C_{38} and C_{37} make up the final interactions as the peptide exits the groove; H54 Hβ and the C_{37} and C_{36} H5 provide information for the final spacer residue of the Tat^{Fin}; **(c)** U_{63} 2′ hydroxyl and K51 Hε indicate a hydrogen bonding interaction; **(d)** R56, R52 Hδ of Tat^{G} and the C_{71}^{+} and U_{63} caps show interaction with ASM_2 and ASM_1; **(e)** R57, R52 Hδ and the C_{75} and U_{40} caps show interaction with ASM_1 and the formation of ASM_3 by R52; **(f)** U_{63} 2′ hydroxyl proton and K51 Hε indicate K51 hydrogen bond with the U_{63} ribose ring.

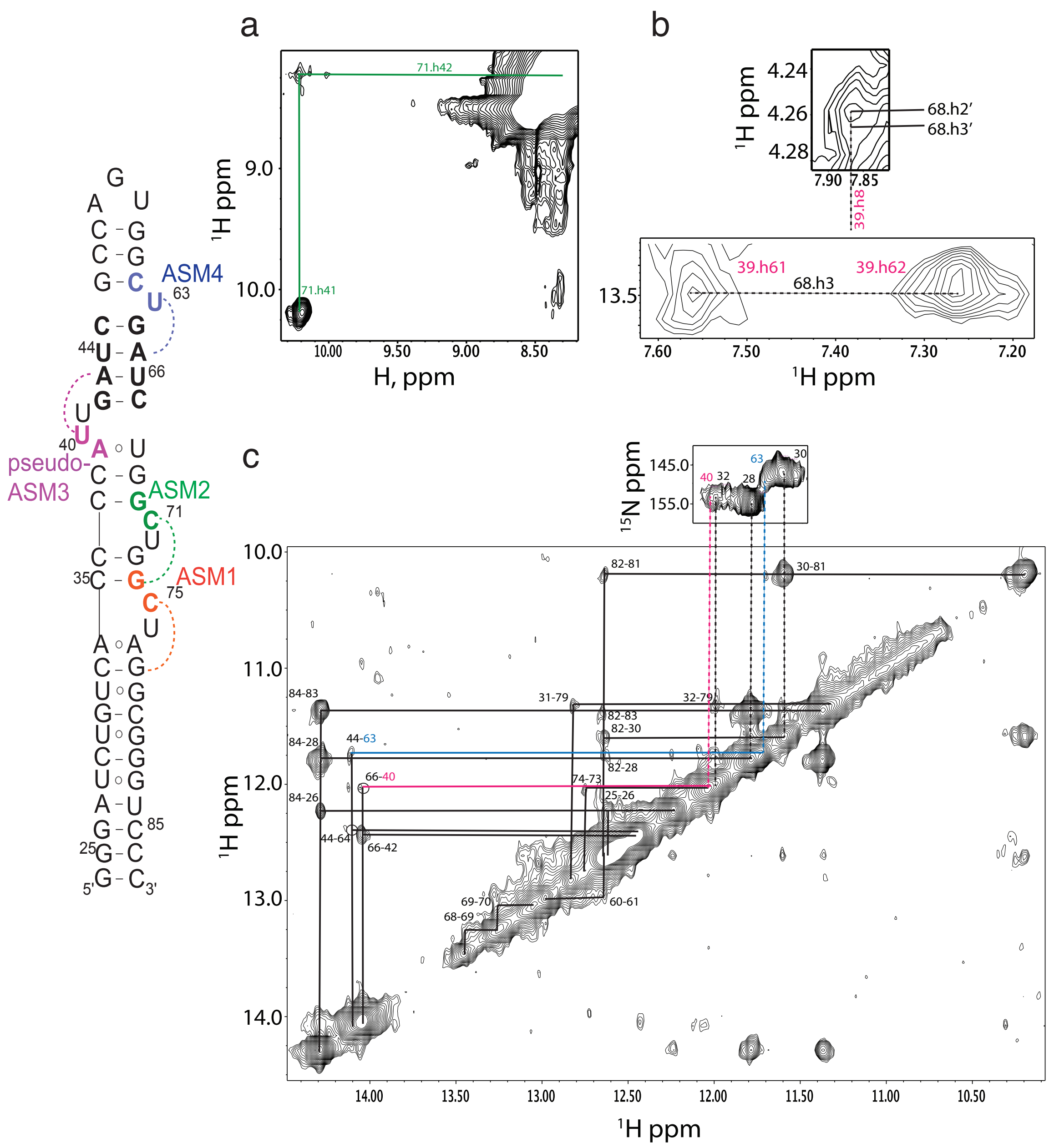

Supplementary Figure 8. Characterization of the A_{39}-U_{68} base pair:
Portions of the ^{1}H– ^{1}H 2D NOESY spectra. **(a)** The C_{71}^{+} amino protons are downshifted due to their participation in a triple base interaction. **(b)** NOEs between the U_{68} imino proton with the A_{39} amino protons and the U_{68} H2′ and H3′ protons with the A_{39} H8 proton confirm that the A_{39}-U_{68} base pair is in a cis Hoogsteen/sugar interaction. **(c)**. Portion of the ^{1}H– ^{15}N 2D HSQC spectrum for ^{15}N, ^{13}C-labeled 7SK-SL1^{apical} supports the assignments of the U_{40} and U_{63} cap imino protons.

**Complex**	**N-value**	**K_d**
HEXIM1:7SK-SL1^{Full}	2.1 ± 0.2	209 ± 30 nM^{*+}
HEXIM1:7SK-SL1$^{Full\text{-}AGU}$	2 ± 0.07	200.0 ± 20 nM*
HEXIM1:7SK-SL1$^{apical\text{-}AGU}$	1.8 ± 0.03	206 ± 60 nM$^{+\infty}$
HEXIM-N-ARM:7SK-SL1^{apical}	1 ± 0.1	229 ± 20 nM$^{\infty£¢}$
Tat-Finland-RBD:7SK-SL1^{apical}	1 ± 0.02	172 ± 10 nM$^{£\pi}$
Tat-Subtype-G-RBD:7SK-SL1^{apical}	1.1 ± 0.1	81 ± 10 nM$^{¢\pi}$

*p=0.9990

$^{+}$p=0.9999

$^{\infty}$p=0.9354

$^{£}$p=0.2797

$^{¢}$p=0.0008

$^{\pi}$p=0.0324

One-way ANOVA, Tukey's multiple comparisons test

Supplementary Table 1. ITC derived binding parameter:
